# 74 Secondary Immunohistochemical Analysis of Viable Cells as Determined by Hematoxylin and Eosin Staining

**DOI:** 10.1093/jbcr/irad045.048

**Published:** 2023-05-15

**Authors:** Bonnie Carney, Cameron D'Orio, Jeffrey Shupp, Jeffrey Carter, William Hickerson, James Holmes, Herbert Phelan

**Affiliations:** Firefighters' Burn and Surgical Research Laboratory, Medstar Health Research Institute, Georgetown University Medical Center, Washington, DC; Firefighter's Burn and Surgical Research Laboratory, MedStar Health Research Institute, Washington, DC; Firefighters' Burn and Surgical Research Laboratory, MedStar Health Research Institute, Georgetown Univeristy School of Medicine, Washington, DC; University Medical Center New Orleans & LSUHSC, River Ridge, Louisiana; Access Pro Medical, LLC, Augusta, Georgia; WFSOM, Winston-Salem, North Carolina; LSUHSC New Orleans Department of General Surgery, New Orleans, Louisiana; Firefighters' Burn and Surgical Research Laboratory, Medstar Health Research Institute, Georgetown University Medical Center, Washington, DC; Firefighter's Burn and Surgical Research Laboratory, MedStar Health Research Institute, Washington, DC; Firefighters' Burn and Surgical Research Laboratory, MedStar Health Research Institute, Georgetown Univeristy School of Medicine, Washington, DC; University Medical Center New Orleans & LSUHSC, River Ridge, Louisiana; Access Pro Medical, LLC, Augusta, Georgia; WFSOM, Winston-Salem, North Carolina; LSUHSC New Orleans Department of General Surgery, New Orleans, Louisiana; Firefighters' Burn and Surgical Research Laboratory, Medstar Health Research Institute, Georgetown University Medical Center, Washington, DC; Firefighter's Burn and Surgical Research Laboratory, MedStar Health Research Institute, Washington, DC; Firefighters' Burn and Surgical Research Laboratory, MedStar Health Research Institute, Georgetown Univeristy School of Medicine, Washington, DC; University Medical Center New Orleans & LSUHSC, River Ridge, Louisiana; Access Pro Medical, LLC, Augusta, Georgia; WFSOM, Winston-Salem, North Carolina; LSUHSC New Orleans Department of General Surgery, New Orleans, Louisiana; Firefighters' Burn and Surgical Research Laboratory, Medstar Health Research Institute, Georgetown University Medical Center, Washington, DC; Firefighter's Burn and Surgical Research Laboratory, MedStar Health Research Institute, Washington, DC; Firefighters' Burn and Surgical Research Laboratory, MedStar Health Research Institute, Georgetown Univeristy School of Medicine, Washington, DC; University Medical Center New Orleans & LSUHSC, River Ridge, Louisiana; Access Pro Medical, LLC, Augusta, Georgia; WFSOM, Winston-Salem, North Carolina; LSUHSC New Orleans Department of General Surgery, New Orleans, Louisiana; Firefighters' Burn and Surgical Research Laboratory, Medstar Health Research Institute, Georgetown University Medical Center, Washington, DC; Firefighter's Burn and Surgical Research Laboratory, MedStar Health Research Institute, Washington, DC; Firefighters' Burn and Surgical Research Laboratory, MedStar Health Research Institute, Georgetown Univeristy School of Medicine, Washington, DC; University Medical Center New Orleans & LSUHSC, River Ridge, Louisiana; Access Pro Medical, LLC, Augusta, Georgia; WFSOM, Winston-Salem, North Carolina; LSUHSC New Orleans Department of General Surgery, New Orleans, Louisiana; Firefighters' Burn and Surgical Research Laboratory, Medstar Health Research Institute, Georgetown University Medical Center, Washington, DC; Firefighter's Burn and Surgical Research Laboratory, MedStar Health Research Institute, Washington, DC; Firefighters' Burn and Surgical Research Laboratory, MedStar Health Research Institute, Georgetown Univeristy School of Medicine, Washington, DC; University Medical Center New Orleans & LSUHSC, River Ridge, Louisiana; Access Pro Medical, LLC, Augusta, Georgia; WFSOM, Winston-Salem, North Carolina; LSUHSC New Orleans Department of General Surgery, New Orleans, Louisiana; Firefighters' Burn and Surgical Research Laboratory, Medstar Health Research Institute, Georgetown University Medical Center, Washington, DC; Firefighter's Burn and Surgical Research Laboratory, MedStar Health Research Institute, Washington, DC; Firefighters' Burn and Surgical Research Laboratory, MedStar Health Research Institute, Georgetown Univeristy School of Medicine, Washington, DC; University Medical Center New Orleans & LSUHSC, River Ridge, Louisiana; Access Pro Medical, LLC, Augusta, Georgia; WFSOM, Winston-Salem, North Carolina; LSUHSC New Orleans Department of General Surgery, New Orleans, Louisiana

## Abstract

**Introduction:**

Accurate clinical differentiation of deep partial-thickness (DPT) and full-thickness (FT) wounds is essential in justifying appropriate treatments for heterogeneous burns. Only recently has non-invasive burn depth imaging been ground-truthed with tissue punch biopsies; an endeavor that will no doubt improve AI imaging algorithms in the near future. Prior literature supports unique apoptotic and necrotic properties of DPT and FT wounds, suggesting a use for not only standard stains (H&E), but apoptotic marker staining in assisting clinical diagnosis of burn depth, progression and/or conversion.

**Methods:**

The aim of this study was to evaluate the presence of apoptotic cells in DPT and FT samples previously diagnosed by a burn biopsy algorithm (BBA-V2) for burn depth and severity by expert review of H&E-stained slides. Eight paired biopsies were collected from DPT and FT wounds in four patients within 72 hours of thermal injury and were immunofluorescently labeled for apoptosis markers by detection of deoxynucleotidyl transferase-mediated dUTP nick end labeling (TUNEL) and cleaved caspase-3 (CC-3) staining. Fluorescence intensity in dermal appendages within images was used to quantify signal at 40x magnification for CC-3. % positive TUNEL cells were graded by an investigator blinded to the burn severity. A higher grade indicated higher % positivity. TUNEL grading was evaluated by Chi square test. CC-3 intensity was evaluated by Student’s t-test.

**Results:**

Across all four patients, FT burns had higher levels of CC-3 in dermal appendages compared to DPT burns (Cy3:DAPI ratios: 1.53 ± 0.28 vs. 0.75 ± 0.13; p=0.1074). Within each patient, all FT biopsy sites had significantly higher levels of CC-3 versus DPT biopsy sites (p< 0.05)(Table). DPT burns had a greater proportion of patients with increasing grades of TUNEL positive cells (DPT: Grade 1: 16%, Grade 2: 36%, Grade 3: 48%; FT: Grade 1: 42.3%, Grade 2: 23.1%, Grade 3: 34.6%, p=0.1178).

**Conclusions:**

CC-3 and TUNEL levels within dermal appendages align with burn depth classification by H&E staining, wherein appendages graded as necrotic had higher levels of CC-3 and lower levels of TUNEL. Therefore, CC-3 and TUNEL are potential secondary histological markers for burn depth. Further analysis of isolated, individual dermal appendages is underway to identify what additional information this secondary marker may add to the diagnosis of burn depth.

**Applicability of Research to Practice:**

For the first time there is access to a large tissue repository of biopsies from burn wounds. Although staining from these samples has been used to build diagnosis algorithms with success, much more can be learned from the cellular pathophysiology when secondary analyses are performed. These assays may help us understand the processes of burn conversion.

